# Generation of Monoclonal Cultures from *Wolbachia*-infected *Drosophila melanogaster* JW18 Cell Line

**DOI:** 10.3791/68207

**Published:** 2025-06-27

**Authors:** Navina Mable Francis, Ewa Chrostek

**Affiliations:** 1 Institute of Environmental Sciences, Faculty of Biology, Jagiellonian University, Krakow, Poland; 2 Jagiellonian University, Doctoral School of Exact and Natural Sciences, Krakow, Poland; 3 Department of Evolution, Ecology and Behaviour, Institute of Infection, Veterinary and Ecological Sciences, University of Liverpool, UK

## Abstract

Cell lines are widely used models in biological research. They are particularly useful in studies of intracellular bacteria that cannot be efficiently propagated outside host organisms. *Wolbachia*, an intracellular symbiont of many invertebrates, induces strong reproductive and antiviral effects in its insect hosts. *Wolbachia*-infected insect cells have been widely used to study *Wolbachia* phenotypes. However, these cell lines often consist of mixed populations of cells with potentially varying phenotypes and responses to experimental treatments. In particular, cell lines with *Wolbachia* have been reported to exhibit variable growth rates and variable *Wolbachia* infection prevalence from one passage to the next. To remedy this, we generated monoclonal cell lines from the *Wolbachia*-infected *Drosophila melanogaster*-derived JW18 cell line. These clonal lines were established at different timepoints and show different *Wolbachia* infection statuses. This variability suggests that any treatment applied to a parental JW18 population could lead to the selection of sub-populations as opposed to influencing the physiology of the entire culture. Here, we present a protocol for generating single-cell clones and continuous clonal cultures from *Wolbachia*-infected insect cells, enabling more controlled and reproducible experiments.

## Introduction

*Drosophila melanogaster* cells are widely used in genetic studies, disease research, and as a platform for high-throughput drug screening^1^. They are also helpful in studying one of the most prevalent intracellular microbes on the planet: *Wolbachia. Wolbachia* is a gram-negative obligate intracellular bacterium found in 40–70% of insect species^2–4^, where it spreads maternally and induces reproductive manipulations^5–8^. *Wolbachia* can also provide infected hosts with antiviral protection^9–15^ which together with reproductive manipulations is being exploited to control arbovirus transmission from mosquitoes to humans^16,17^. In various systems, *Wolbachia* has been shown to control apoptosis^18^, supply essential nutrients^19^, and alter gene and protein expression^20–25^.

Cell lines emerged as a model to study *Wolbachia* because of their simple biology, convenience, and ability to propagate in large quantities. Cell lines have proven useful in pre-adapting *Wolbachia* to symbiosis with novel hosts^26^ and allowing *Wolbachia* to accumulate mutations^27,28^. They were used to screen for anti-*Wolbachia* compounds, which can be helpful in the treatment of filarial diseases^29^ and studying *Wolbachia*-host translation interaction^30^. Although cell lines are indispensable in studies on *Wolbachia* symbioses^29–31^, the mechanisms underlying most *Wolbachia* phenotypes remain unknown. This suggests that the cell culture model system can still be improved.

*Wolbachia*-infected insect cell lines are derived from insect embryos and larvae. Aa23, derived from *Aedes albopictus* embryos, was the first stable *Wolbachia*-infected line^32^, subsequently used for many studies on *Wolbachia* symbiosis^33–35^. The JW18 cell line was one of the first naturally *Wolbachia*-infected lines generated from *D. melanogaster*^29,36^. This line, derived from 1 h to 15 h embryos of *w*Mel-infected flies, has also served as a source of *Wolbachia* for other *Drosophila* cell lines: 1182-4H and S2R+^37^. As embryos have a high proportion of pluripotent cells, by adapting to grow in plastic, they can acquire new characteristics, sometimes different for individual cell lineages. This may explain the heterogeneity of cell morphologies and *Wolbachia* infection frequencies observed in these cells at any one time and over generations^29^.

The cellular diversity within the JW18 culture raises concerns, as studies on mosquito cell lines, such as Aag2, have shown that monoclonal and parental cell lines may respond differently to treatments due to variations in cell characteristics^38^. Consequently, administering treatments to a mixed cell population could yield misleading results. To study the response of a homogenous cell population to a treatment (not selection for individual cell types) we have generated monoclonal cultures from the parental JW18 cell line. There have been no previous attempts made to generate monoclonal cultures from these cells. The protocol involves isolating single cells from the parental JW18 cell line, followed by their maintenance and transfer to flasks to establish continuous clonal cultures. We checked the *Wolbachia* infection status of individual lines, showing that they differ in this key characteristic. Homogeneous cell cultures are critical in ensuring the accuracy and reproducibility of future experiments.

## Protocol

NOTE: All work with live cells and their media must be performed in a biological safety cabinet to avoid contamination.

### 1 Media preparation

1.1. Prepare fresh Culture Medium by supplementing Shield and Sang Insect medium with 10% fetal bovine serum (FBS).

1.2. Spent and conditioned media

1.2.1. Grow JW18 cells in a 25 cm^2^ flask containing fresh Shield and Sang Insect medium supplemented with 10% FBS at 25 °C. Incubate until the cells reach confluency (~1 week).

1.2.2. Filter the 1 week-old spent cell culture medium used to grow the cells, to remove *Wolbachia*, cell debris, and other contaminants: use a 5 μm sterile filter and then a 0.2 μm sterile filter fitted to a 50 mL syringe.

1.2.3. Prepare conditioned medium by supplementing fresh medium with 20% of spent medium. Prepare enough conditioned medium for one 96-well plate (~30 mL); using serological pipette, mix 6 mL of the filtered spent medium with 24 mL of fresh Shield and Sang Insect medium supplemented with 10% FBS.

### 2 Preparation of the 96-well plate

2.1. Preparation of JW18 cell suspension

2.1.1. Scrape the cells from a confluent, 1-week-old flask using a cell scraper and resuspend them using a serological pipette.

2.1.2. Count the cells using a Neubauer chamber and dilute them with conditioned medium to achieve a final concentration of 2 × 10^3^ cells/mL.

2.2. Adding conditioned medium to the plate

2.2.1. Transfer the prepared conditioned medium to a reagent reservoir.

2.2.2. Using a multichannel pipette, add 100 μL of conditioned medium to each well of the 96-well plate, except well A1.

2.3. Serial dilution of the cell suspension

2.3.1. Add 200 μL of the cell suspension to well A1.

**Figure 1:**
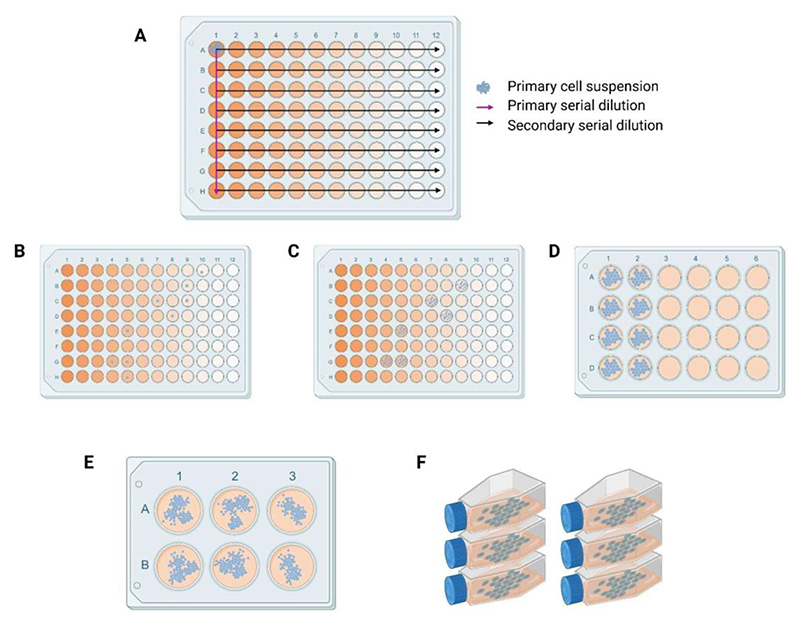
Workflow for monoclonal culture generation. (**A**) Primary and secondary dilutions in the 96-well plate. (**B**) Single cells post serial dilution. (**C-E**) Progression in multiplication of cells and transfer from 96-well to 24- and 6-well plate, respectively. (**F**) Continuous cultures in 25 cm^2^ cell culture flasks.

2.3.2. Using a single-channel pipette, transfer 100 μL from well A1 to well B1 and mix gently (avoid bubbles) as in Figure 1A.

2.3.3. Repeat the serial dilution down column 1, discarding the final 100 μL from the last well.

2.4. Dilution across the plate

2.4.1. Add 100 μL of conditioned medium to each well in the first column, to a total volume of 200 μL. Mix gently.

2.4.2. Using the multichannel pipette, transfer 100 μL from the first column to the second. Mix gently.

2.4.3. Repeat this process for all columns (1–12) across the plate (Figure 1A).

2.4.4. After resuspension, discard 100 μL from the last column. At this point, each well contains 100 μL of cell suspension.

2.4.5. Add an additional 100 μL of culture medium to all wells, bringing the final volume in each well to 200 μL.

### 3 Identification of single cells

3.1. Cover the plate, seal it with Parafilm, and label it appropriately.

3.2. Observe the plate under a microscope, checking each well carefully for single cells (Figure 1B). Be sure to scan the wells and the edges thoroughly to confirm the presence of only one cell.

NOTE: This is one of the most critical steps in the protocol.

3.3. Record the well number of single cells on a 96-well plate map (on paper).

NOTE: Single cells are more likely to be found in wells toward the right and bottom of the plate.

3.4. Once single cells are identified and marked, store the plate in an incubator at 25 °C. Monitor the cells every 2 days.

### 4 Maintenance and transfer of cells

4.1. Cell monitoring

**Figure 2:**
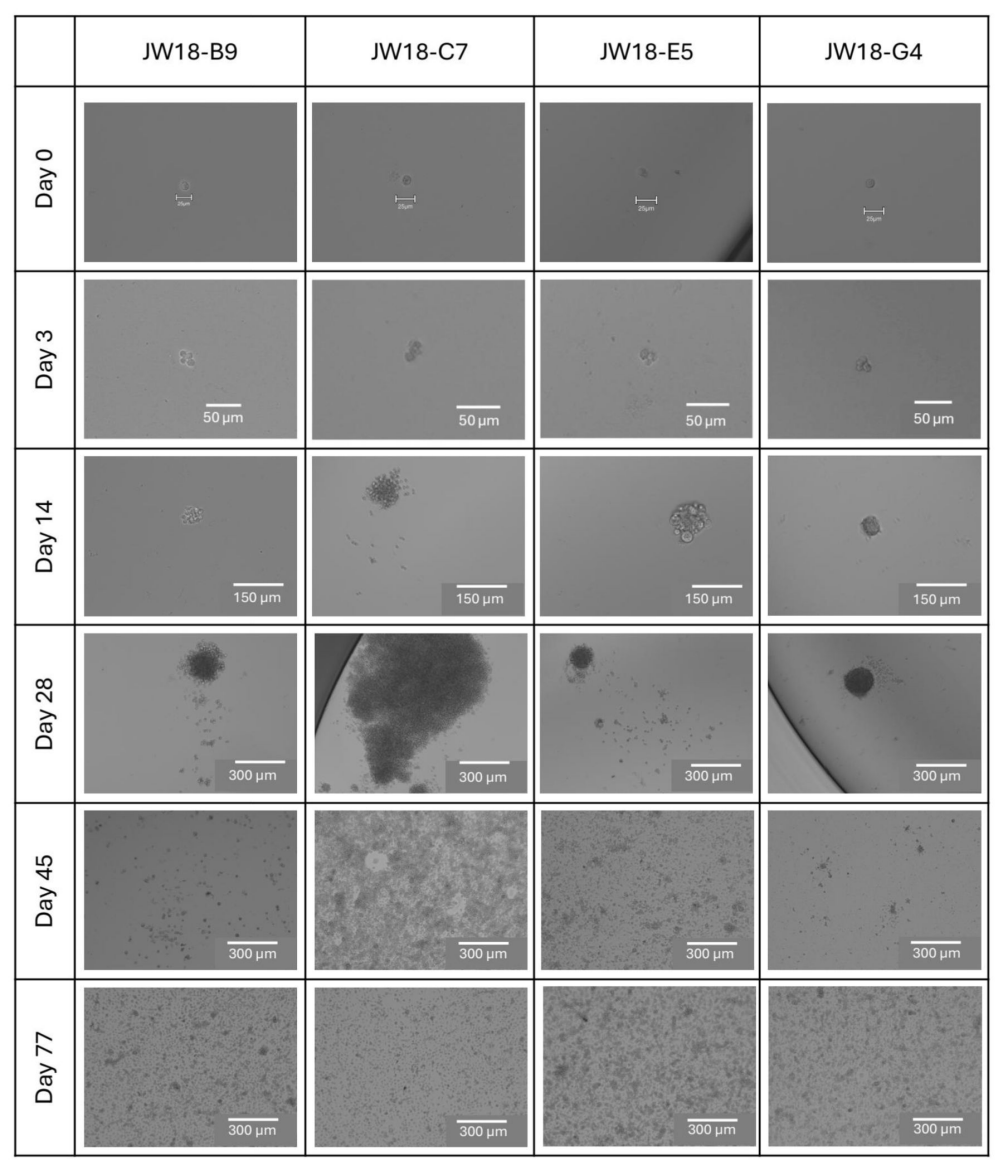
Progression of JW18 clonal lines from single cell to multicellular culture. Brightfield images of clonal lines from day 0 (day of plating) to day 77, when all lines were successfully established in a 25 cm^2^ cell culture flask. Scale bars = 25 μm (day 0), 50 μm (day 3), 150 μm (day 14), 300 μm (days 28, 45, 77).

NOTE: JW18 cells typically take 3 days to start dividing from a single cell, and clonal populations may grow at different rates (Figure 2).

4.1.1. Monitor the wells every 2–3 days for bacterial contamination and to ensure the medium has not dried out. If the wells appear to be drying out, top them up with fresh medium as required.

4.2. Cell expansion

4.2.1. Once cells reach confluence in the 96-well plate, transfer them to a 24-well plate (Figure 1D).

4.2.2. Subsequently, transfer the cells to a 6-well plate and finally to a flask (Figure 1E, F).

NOTE: This stepwise expansion increases the likelihood of establishing a continuous clonal cell line.

4.3. Cell freezing

4.3.1. Once a clonal culture is established, freeze a portion of the cells for future use.

4.3.1.1. Scrape the cells from the flask using a cell scraper and transfer them to a 15 mL centrifuge tube. Centrifuge at 250 × *g* for 5 min at 4 °C.

4.3.1.2. Prepare a 2x freezing medium (Shield and Sang Insect Medium supplemented with 20% FBS and 10% DMSO).

4.3.1.3. After centrifugation, discard part of the supernatant, leaving half of the final desired volume. Add an equal volume of the 2x freezing medium to the remaining supernatant and gently resuspend the pellet using a pipette.

4.3.1.4. Aliquot 1 mL of the suspension into each 1.5 mL cryovial and place the vials on ice. Store the vials at −80 °C overnight, then transfer them to a liquid nitrogen tank the next day for long-term storage.

### 5 PCR to confirm *Wolbachia* status

5.1. Extract DNA from the monoclonal culture samples using the phenol-chloroform method^39^.

5.1.1. Place 1 mL of confluent scraped and resuspended cells in medium in a 1.5 mL microcentrifuge tube and centrifuge the cells at 290 × *g* for 5 min at 4 °C. Discard the supernatant (cell culture medium) and place the tube on ice.

5.1.2. Add 250 μL of Solution A, which consists of 0.1 M Tris HCl (pH 9.0), 0.1 M EDTA, and 1% SDS. Resuspend the cells and incubate at 70 °C for 30 min in a heating block.

5.1.3. After incubation, add 35 μL of 8 M potassium acetate, shake the tube gently (avoid vortexing), and incubate on ice for another 30 min.

5.1.4. Spin at 13,000 rpm for 15 min at 4 °C, transfer the supernatant to a new tube, and add an equal volume of phenol-chloroform. Shake well and spin again, repeating this step once.

5.1.5. Transfer the supernatant to a new tube, then add 150 μL of isopropanol, shake, and spin at 10,000 rpm for 5 min. Remove the supernatant carefully, wash the pellet with 1 mL of 70% ethanol, and spin at 13,000 rpm.

5.1.6. After drying the pellet for 10 min at room temperature, resuspend it in 100 μL of TE buffer. Quantify the DNA and store at -20 °C.

5.2. Perform a conventional PCR with the DNA extracted from the monoclonal culture samples to detect *Wolbachia* using DreamTaq Green PCR master mix. Use *wsp* 81F and *wsp* 691R primers^40^ for *Wolbachia* and *Drosophila rpl32* primers^41^ for DNA quality control.

5.2.1. Add 7.5 μL of master mix, 0.5 μL of each primer at 10 μM, 1 μL of DNA diluted 1:50, and 5.5 μL of molecular grade water to get 15 μL of the reaction mixture.

5.2.2. Use the following PCR program: 94 °C for 4 min, 30 cycles x (94 °C for 40 sec, 55 °C for 40 sec, 72 °C for 30 sec), 72 °C for 10 min. Post PCR, save samples at 4 °C.

5.3. Carry out Agarose gel electrophoresis in a 2% agarose gel in TAE buffer at 90 V with the obtained PCR products to visualize the bands. Add a DNA ladder alongside to identify the bands for *wsp* (632 bp) and *rpl32* (194 bp).

### 6 FISH staining to visualize *Wolbachia* infection in cell lines

6.1. Perform fluorescent *in situ* hybridization (FISH) as described in Stellaris FISH protocol for adherent cells to stain *Wolbachia* with Quasar 670-tagged 16s rRNA FISH probe set. Stain the nucleic acids with Hoechst 33342.

NOTE: This probe set was designed based on *w*Mel 16S rDNA sequence and matches the probe set published in Schneider et al. ^42^ (see Supplemental Table S1).

6.1.1. Prepare reagents (Fixation buffer, Hybridization buffer, Wash buffer A and B, reconstituted custom probe set) following the manufacturer’s instructions^43^.

6.1.2. Grow adherent cells on 70% ethanol-sterilized 18 mm round cover glass in 24-well culture plate. Once the cells reach the desired confluency, aspirate the growth medium and wash with 1 mL of 1x PBS. Add 1 mL of fixation buffer and incubate at room temperature for 10 min. Wash the cells twice with 1 mL of 1x PBS.

6.1.3. Permeabilize by immersing the coverslip with cells in 1 mL of 70% ethanol and incubate overnight at 4 °C; store the samples in ethanol at this temperature for up to 1 week before hybridization. Aspirate the ethanol and add 1 mL of Wash Buffer A; incubate at room temperature for 2–5 min.

6.1.4. Prepare a humidified chamber by placing a water-saturated paper towel and a layer of Parafilm in a 150 mm culture dish. Pipette 100 μL of hybridization buffer containing 1 μL of 12.5 μM probe stock onto the Parafilm. Gently invert the cover glass, placing cells side down onto the drop of hybridization buffer. Cover and seal the chamber with Parafilm, then incubate in the dark at 37 °C for at least 4 h (up to 16 h if needed).

6.1.5. After hybridization, carefully transfer the cover glass cells side up to a fresh well containing 1 mL of Wash Buffer A and incubate at 37 °C for 30 min. Aspirate the buffer and add 0.5 μL of 20 mM Hoechst stock to 700 μL of Wash Buffer A; incubate at 37 °C for 30 min in the dark.

6.1.6. Remove the Hoechst solution and add 1 mL of Wash Buffer B; incubate at room temperature for 2–5 min. Finally, place a small drop (10 μL) of mounting medium on a microscope slide and mount the cover glass cells side down onto the drop. Gently wick away excess mounting medium and seal the perimeter of the cover glass with clear nail polish.

6.1.7. Store the slides at 4 °C and proceed to imaging as soon as possible.

6.1.8. Process the images using ImageJ^44^. Split channels and change the color palette as required.

## Representative Results

**Figure 3:**
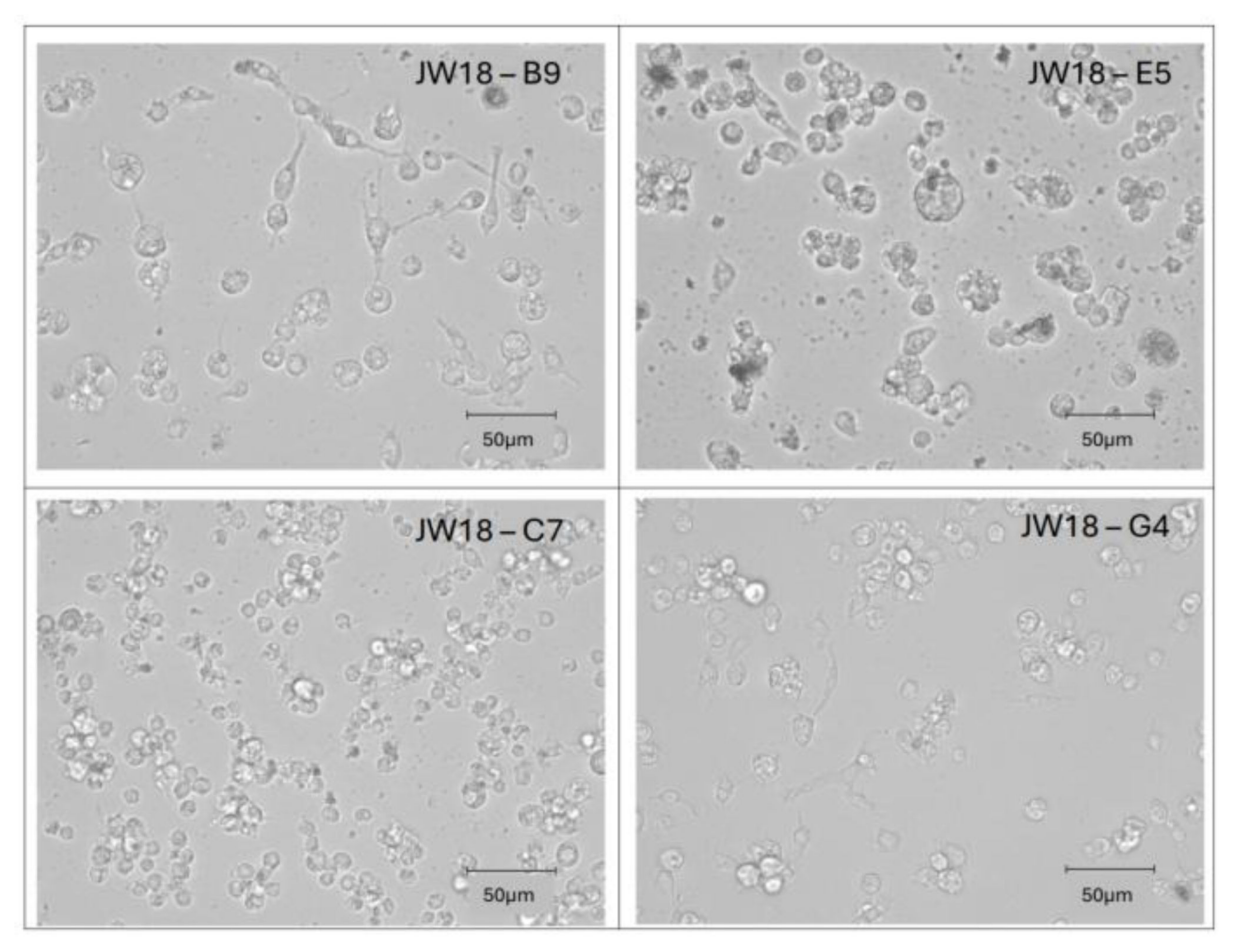
Morphology of individual JW18 clonal lines. Brightfield images of clonal lines showing cell morphologies at passage 30 post establishment. Scale bars = 50 μm.

We visualized the process of establishing monoclonal cultures with light microscopy from the day of preparing the single-cell suspension and plating (day 0) to establishing of continuous cell lines (day 45 or 77, Figure 2). The first signs of cell division were observed on day 3 (4 days post plating, as plating was performed on day 0) when there were already four cells per well (Figure 2). On day 14, the clones grew either as a monolayer (JW18-B9, C7, and E5) or a sphere (JW18-G4), which was disrupted during passage. On day 28, the differences in the growth between cell lines were most obvious. JW18-C7 culture was confluent on day 45, while the remaining clonal cultures were only established at day 77. We have also visualized the four different cultures 30 passages post establishment to capture differences in cell morphologies (Figure 3).

**Figure 4:**
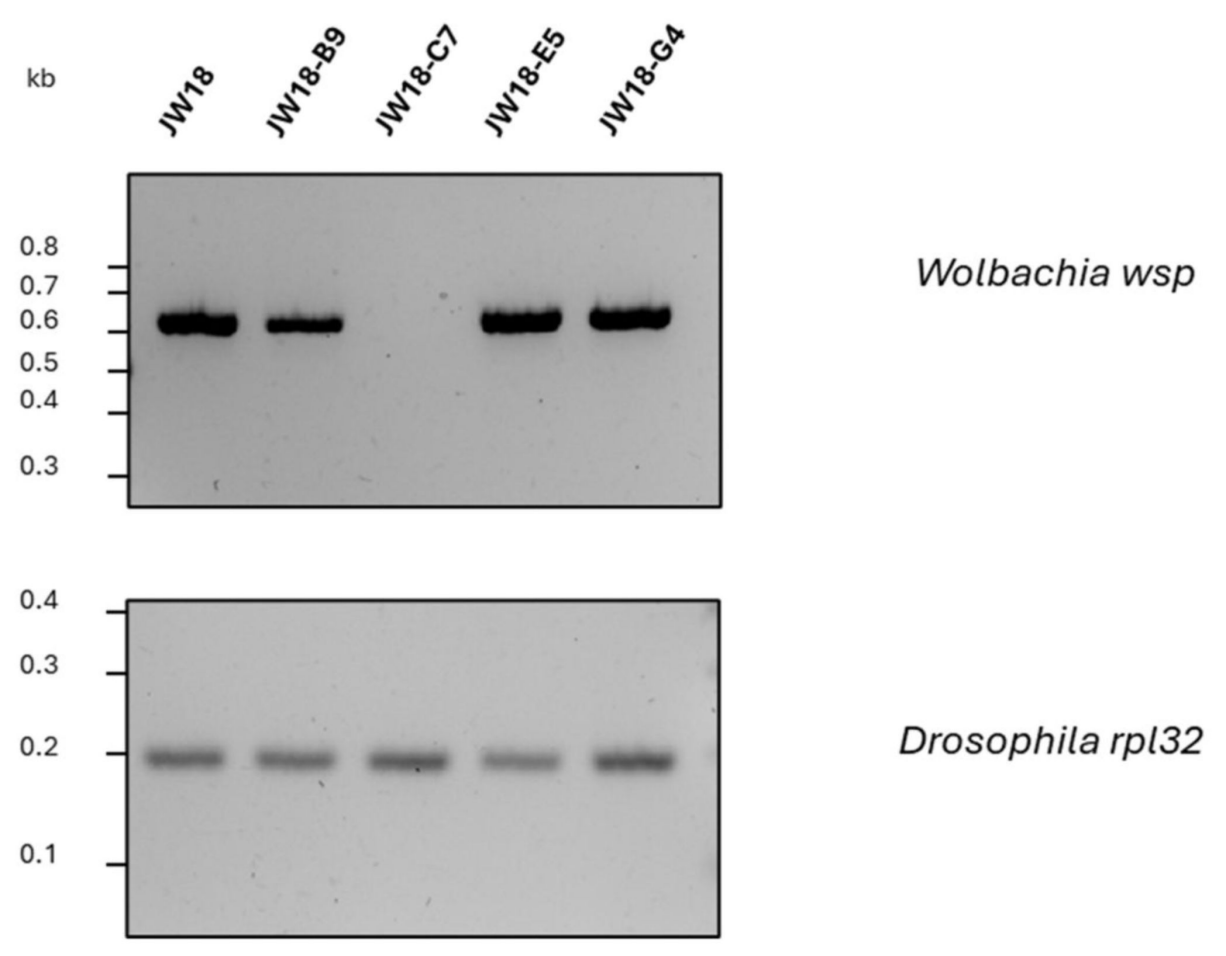
*Wolbachia* status of clonal cultures examined by PCR. PCR for *Wolbachia* in JW18 parental cell line and clonal lines JW18-B9, JW18-C7, JW18-E5, and JW18-G4 at passage 11 post establishment. *Wolbachia wsp* band confirms the presence of *Wolbachia*, while *Drosophila rpl32* band is a DNA quality control PCR.

**Figure 5:**
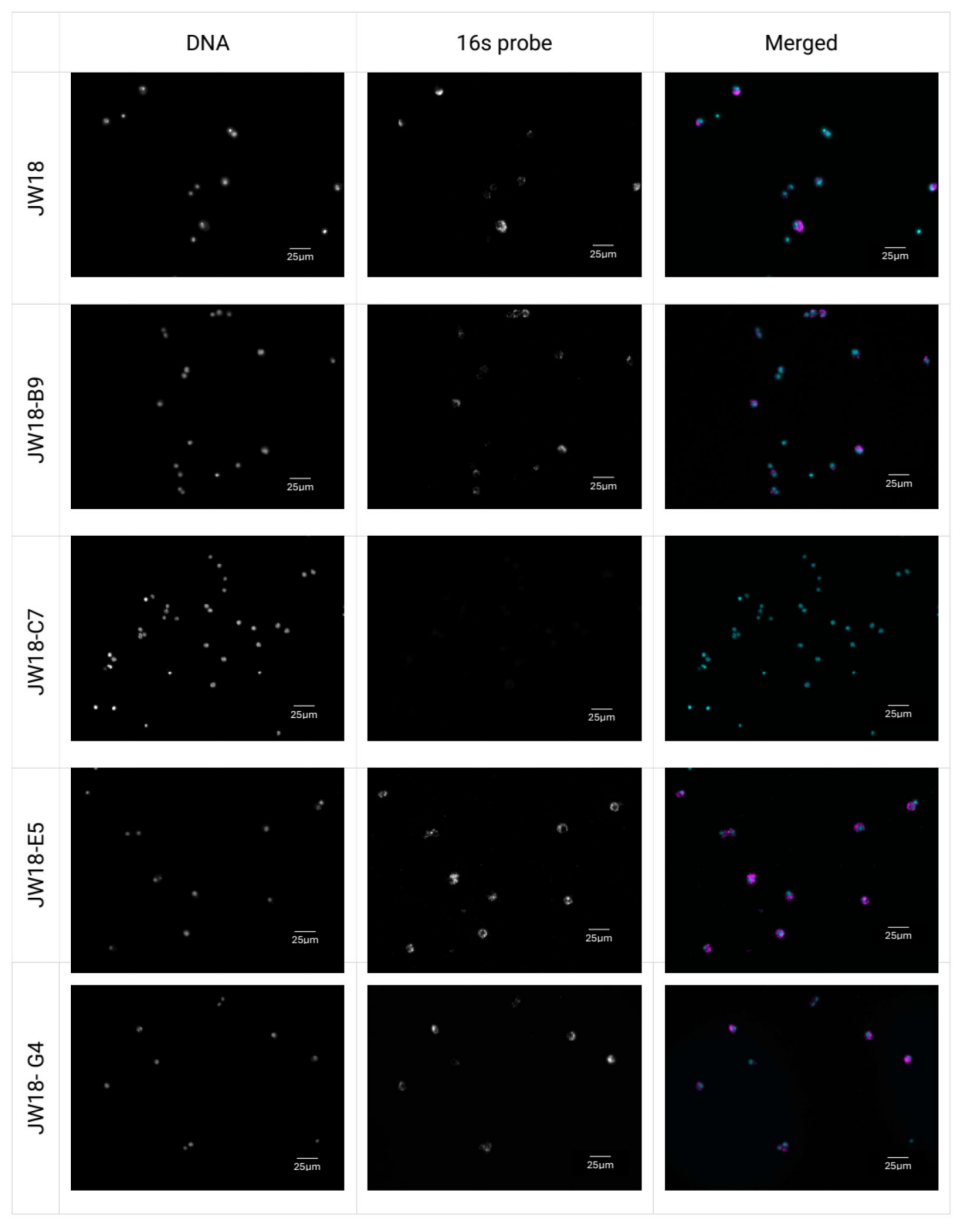
*Wolbachia* status of clonal cultures examined by FISH. JW18 parental cell line and clones JW18-B9, JW18-C7, JW18-E5, JW18-G4 visualized by FISH staining at passage 13 post establishment. *Wolbachia* is stained with Quasar 670-tagged 16s rRNA Stellaris FISH probe set in magenta, and nucleic acids are stained with Hoechst 33342 in cyan. Scale bars = 25 μm.

To determine *Wolbachia* infection status of the monoclonal cultures, we conducted PCR (Figure 4) and FISH (Figure 5). We observed that all but one clone (JW18-C7) produced a band for *wsp* gene, indicative of a *Wolbachia* infection. FISH corroborated the PCR results: clones JW18-B9, JW18-E5, and JW18-G4 retained *Wolbachia* infection, while the clone JW18-C7 did not have *Wolbachia*. Overall, from a mixed JW18 cell population, we were able to establish four clonal cultures, three of which retained the original *Wolbachia* infection. The raw data can be accessed at Figshare: https://figshare.com/s/15fde47ca7338f31dda9.

## Discussion

Here we outline a standardized method for generating single-cell clones from the JW18 *D. melanogaster* cell line, which can be applied to other *Drosophila* and insect cell lines. The protocol involves serial dilution of a prepared cell suspension across a 96-well plate to isolate individual cells. These cells are then incubated under optimal conditions to establish continuous cultures. The protocol also includes the addition of 20% conditioned media to promote cell survival and single-cell division^45,46^.

A critical step in the protocol is verifying the presence of a single cell in each well following serial dilution. This step requires meticulous attention, as it is essential for the success of the entire protocol. To facilitate future confirmation of the single-cell origin, capturing images of the selected wells from various angles is recommended.

Another key step involves transferring cells between culture dishes. To ensure the successful establishment of a continuous culture, cells should only be transferred once they have reached confluence. If cell growth stagnates for more than two weeks, refreshing the media can promote further growth. Premature transfer of cells before confluence may result in cell death.

While this protocol is more time-consuming and labor-intensive than other commonly used methods, such as Fluorescence-Activated Cell Sorting (FACS)^38,46^, it offers several advantages. These include cost-effectiveness, minimal risk of cell damage, no requirement for fluorescent markers, expensive equipment, and optimization time. Overall, this protocol is well-suited for generating homogeneous insect cell populations from continuous cell lines.

### Properties of the monoclonal cultures

Our results show that the monoclonal cultures differ in terms of speed of their establishment and *Wolbachia* infection status (Figure 2, Figure 4, and Figure 5). *Wolbachia*-free clone JW18-C7 was the fastest one to reach confluence in our single-cell isolation protocol. *Wolbachia* could either have always been absent from this cell lineage, or it could have been lost due to the inability to keep up with the division rate of the host cell. It could also have been lost due to the initial low cell density in our cloning protocol. However, as this did not happen for the other clonal cell lines, we consider this unlikely. The experimental infection of JW18-C7 with *Wolbachia* (alongside other, tetracycline-treated clones) could answer whether this line is permissive to the symbiont.

The cellular heterogeneity is not unique to the JW18 cell line and has been reported before for other *Wolbachia*-infected cell lines^32,47–52^. A previous attempt at its amelioration involved a transfer of *Wolbachia* from *D. simulans* eggs or Aa23 cells to a C7-10 *A. albopictus* cell line^53^. C7-10 has been cloned many years prior to *Wolbachia* transfers^54,55^, and the transfers do not constitute the natural infection we intend to study.

The difference in *Wolbachia* infection status and time of monoclonal culture establishment (indicative of differences in cell division rate) for individual JW18 clones can have profound implications for previous studies linking *Wolbachia* titer and insect cell growth rate in response to specific treatments. Altered cell numbers, symbiont densities, or changes in gene expression upon treatment might result from selection for cell types with specific properties rather than changes in the state of all cells in a mixed population. Administering treatments to a mixed cell population with the variability in key characteristics may hence yield biased, unreproducible results.

## Supplementary Material

Supplemental Table S1

Supplemental Table Legand

## Figures and Tables

**Table 1:** 

Name of Material/Equipment	Company	Catalog Number	Comments/Description
0.22 μm syringe fliter	TPP	99722	Sterile
10X Phosphate Buffered Saline (PBS)	ThermoFisher Scientific	70011044	Sterile
12 or 18 mm round coverglass	Any brand		
15ml Falcon tubes	Genoplast	601052	Sterile
24-well plate	CytoOne	CC7682-7524	Tissue culture treated
5 μm syringe filter	Whatman	10462000	Sterile
6-well plate	CytoOne	CC7682-7506	Tissue culture treated
96-well plate	CytoOne	CC7682-7596	Tissue culture treated
Agarose	ThermoFisher Scientific	J66501.30	
Cell culture flask, 25 cm^2^	Greiner Bio-one	391-3103	Sterile
Cell scrapers	Greiner Bio-one	391-3010	Sterile
Clear nail polish	Any brand		
CryoPure vials	Sarstedt	72.379	Sterile
DMSO, cell culture grade	Merck	D2650-100ML	Sterile
DreamTaq PCR Master Mix	ThermoFisher Scientific	K1081	
EDTA	Merck	324503-100GM	Sterile
Electrophoresis Power Supply	Merck	PS 251-2	
Eppendorf® Concentrator Plus	Merck	EP5305000509	Vaccum concentrator of any brand can be used instead.
Ethanol	Merck	1009861000	
EVOS™ M5000 Imaging System	ThermoFisher Scientific	AMF5000	Inverted microscope of any brand can be used to monitor clone progression. Fluorescent microscope is required to visualise FISH.
Fetal Bovine Serum	Merck	F9665	Heat-inactivated
Formaldehyde	ThermoFisher Scientific	047377.9L	
Formamide	Merck	F7503-100ML	
Gel electrophoresis tanks	Cleaver Scientific	MSMIDI	
Gel Imager	BioRad	12009077	GelDoc Go Gel Imaging System
GeneRuler 100 bp DNA Ladder	ThermoFisher Scientific	SM0243	
Heating Block	ThermoFisher Scientific	88870005	
Herasafe™ 2025 Class II Biological Safety Cabinet	ThermoFisher Scientific	51033314	Biological safety cabinet of any brand can be used as long as it maintains sterile environment inside.
Hoechst 33342 Solution (20 mM) 5 mL	ThermoFisher Scientific	62249	Store in the dark.
Hydrochloric acid	Merck	258148-2.5L	
Integra Biosciences Corp PIPETBOY acu 2 Pipet Aid	Fisher Scientific	NC0085686	Serological pipette controler of any brand can be used.
Isopropanol	Merck	I9516-1L	
Laboratory freezer	Liebherr	LGUex 1500 MediLine	Any freezer capable of maintaining constant temperature is suitable.
Liquid nitrogen tank	ThermoFisher Scientific	CY50985	
Microscope slides	Merck	CLS294875X25-72EA	
Molecular grade water			
Multichannel pipette	Eppendorf	3125000036	
Nanodrop	Peqlab	ND-1000	
Neubauer Hemocytometer	Merck	BR717810-1EA	
Parafilm® M Sealing Film	Merck	HS234526B-1EA	
Peltier-cooled incubator	Memmert	IPP55plus	Any incubator capable of maintaining constant 25°C is suitable. No CO_2_ control is required for JW18 cell line or its clones.
Phenol/Chloroform/Isoamyl alcohol (25:24:1)	ThermoFisher Scientific	327115000	
Pipette set	Eppendorf	EP2231300008	
Potassium acetate	Merck	P1190-500G	Sterile
Quasar 670-tagged 16s rRNA FISH probe set	Biosearch Technologies		Custom made, sequences of the probes can be found in Table 1.
Refrigerated MicroCentrifuge	Labnet	C2500-R	
*rpl32* primer forward 5’-CCGCTTCAAGGGACAGTATC-3’	Eurofins genomics		Custom made based on the reference cited in the paper
*rpl32* primer reverse 5’-CAATCTCCTTGCGCTTCTTG-3’	Eurofins genomics		Custom made based on the reference cited in the paper
SDS	ThermoFisher Scientific	AM9823	Sterile
Serological pipettes	Corning	357771	
Shields and Sang M3 Insect Medium	Merck	S8398	Sterile
Stellaris RNA FISH Hybridization Buffer	Biosearch Technologies	SMF-HB1-10	Sterile
Stellaris RNA FISH Wash Buffer A	Biosearch Technologies	SMF-WA1-60	Sterile
Stellaris RNA FISH Wash Buffer B	Biosearch Technologies	SMF-WB1-20	Sterile
Sterile Reagent Reservoirs	ThermoFisher Scientific	8094	Sterile
TAE buffer	Merck	1061741000	1x
TE buffer	Merck	93283	Sterile
Thermal cycler	Syngen Biotech	Thermoblock 96	Thermal cycler of any brand can be used instead.
Tris base	Merck	10708976001	Sterile
Ultra low temperature freezer	PHC corporation	MDF-DU502VH-PE	Any laboratory freezer capable of maintaining -80 °C can be used.
Vectashield® Mounting Medium	Vector Laboratories	H-1000-10	
*wsp* primer forward 5’-TGGTCCAATAAGTGATGAAGAAAC-3’	Eurofins genomics		Custom made based on the reference cited in the paper
*wsp* primer reverse 5’-AAAAATTAAACGCTACTCCA-3’	Eurofins genomics		Custom made based on the reference cited in the paper

## Summary

Cell lines are useful in studies on intracellular organisms that cannot be cultured outside their host cells. Although *Wolbachia*-infected lines have enabled many discoveries, the heterogeneity within these lines can lead to variable experimental outcomes. We present a protocol for generating single-cell clones and continuous cultures from *Wolbachia*-infected JW18 cells.
