# Supplementary material for: Generation of Monoclonal Cultures from Wolbachia-infected Drosophila melanogaster JW18 Cell Line
Source: J Vis Exp. Author manuscript; Available in PMC 2025 Jul 21. (PMC7617923; doi:10.3791/68207)
Supplement: Supplemental Table Legand [file EMS206779-supplement-Supplemental_Table_Legand.pdf]

Supplemental Table S1: Oligonucleotide probes designed against *Wolbachia* *w*Mel 16S rDNA and their comparison to the probes published by Schneider et al.[42].
